# Microhardness, Indentation Size Effect and Real Hardness of Plastically Deformed Austenitic Hadfield Steel

**DOI:** 10.3390/ma16031117

**Published:** 2023-01-28

**Authors:** Quanshun Luo, Matthew Kitchen

**Affiliations:** Materials and Engineering Research Institute, Sheffield Hallam University, Sheffield S1 1WB, UK

**Keywords:** real hardness, indentation size effect (ISE), Hadfield steel, straining hardening, Nix-Gao model, Meyer’s power law

## Abstract

Microhardness testing is a widely used method for measuring the hardness property of small-scale materials. However, pronounced indentation size effect (ISE) causes uncertainties when the method is used to estimate the real hardness. In this paper, three austenitic Hadfield steel samples of different plastic straining conditions were subjected to Vickers microhardness testing, using a range of loads from 10 to 1000 g. The obtained results reveal that the origin of ISE is derived from the fact, that the indentation load *P* and the resultant indent diagonal *d* do not obey Kick’s law (*P* = *A* · *d*^2^). Instead, the *P* and *d* parameters obey Meyer’s power law ***(****P* = *A · d^n^*) with *n* < 2. The plastically strained samples showed not only significant work hardening, but also different ISE significance, as compared to the non-deformed bulk steel. After extensive assessment of several theoretical models, including the Hays-Kendall model, Li-Bradt model, Bull model and Nix-Gao model, it was found that the real hardness can be determined by Vickers microhardness indentation and subsequent analysis using the Nix-Gao model. The newly developed method was subsequently utilised in two case studies to determine the real hardness properties of sliding worn surfaces and the subsurface hardness profile.

## 1. Introduction

Indentation is the simplest mechanical test with which to measure material hardness. A well-recognised problem in microhardness testing, however, is that the measured hardness value depends strongly on the applied indentation load or indent size, known as the indentation size effect (ISE). In this paper, we present a comprehensive evaluation of the most-used theoretical models to demonstrate a reliable method which can measure the real hardness, i.e., the ISE-independent hardness, by using only small indentation loads in the micro scale. 

The hardness property reflects the ability of a solid material to resist plastic deformation, which is widely used to evaluate the strength and wear resistance of engineering materials. In 1812, Austrian mineralogist Fredrich Mohs was one of the first people to describe a qualitative methodology which can be used to determine the hardness of a material, where a hard material tends to scratch a softer material. By 1856, more quantitative approaches were introduced by William Wade, where a pyramid-shaped hardened tool was used [1]. In 1885, the Kick law was established, which supposes a quadratic relationship between the applied load *P* and the indentation diagonal *d*, as shown in Equation (1) [2]. The Kick law, implying a linear relationship between the indentation load and the resultant indent area, formed the basis of several macrohardness tests, including Brinell hardness, Meyer hardness, Vickers hardness and Knoop hardness [1]. In these macrohardness tests, where the applied indentation load ranges between 0.2–3000 kgf, the hardness value is independent of the indentation load. This brings about great convenience for the applications of hardness tests in the research and industrial manufacturing process control of engineering materials, especially of the Brinell and Vickers hardness tests.

*P* = *K* · *d*^2^ (1)

The Vickers hardness test was developed in the 1920′s, in which a square-based pyramid diamond indenter was designed to have specific geometrical configurations, namely to make opposite sides meet at the apex with an angle of 136°, the edges at 148° and faces at 68°. Moreover, unlike other hardness tests, Vickers hardness test is not only used for macrohardness, but also for microhardness, where the indentation load is less than 0.2 kgf [1,3]. The formula to calculate the Vickers hardness number *H* is shown in Equation (2), where *A* and *d* stand for the actual area and the average projected diagonal length of the resultant indent, respectively, at the indentation load *P*. The units of the *H*, *P*, *A* and *d* are kgf/mm^2^, kgf, mm^2^ and mm, respectively. If the units of *P* and *d* are Newton (N) and micrometer (μm), respectively, Equation (2) is re-written as Equation (3), with *H* in the unit GPa [2].

(2) $$H=\frac{P}{A}=1.8544\;\cdot \;\frac{P}{d^{2}}$$

(3) $$H=1854.4\;\cdot \;\frac{P}{d^{2}}$$

In most cases, the measured hardness value increases with the decrease in the indentation load, or with the decrease in the indent size, which is termed as positive ISE. In fact, the recognition of ISE may be dated back as early as 1908, when Meyer’s power law was deduced, which is an empirical equation to describe the relationship between indentation load *P* and indent diagonal *d*, as shown in Equation (4) [2]. In the equation, the Meyer index *n* is a material-related parameter, which can be experimentally determined to be between 1.5 and 2.5. When Meyer index is equal to 2, Kick law can be applied to pyramid- and cone-shaped hardness indenters, making the hardness independent of the indentation load and the diagonal length. If this is not applied, then the measured hardness would vary with the indentation load. In other words, the existence of ISE means that the microhardness properties of materials can only be compared when they are measured under the same indentation load. Even then, the measured microhardness does not strictly present the real hardness of a material, due to the unknown ISE influence. This becomes a serious problem for samples of micro-scale volumes, such as particles, fibers, thin films and engineered surfaces, where only small indentation loads are allowed to ensure the indentation takes place within the small sample volumes. In these circumstances, the real hardness can be obtained only when the related ISE contribution is removed.

(4) $$P=A\;\cdot \;d^{n}$$

It can be derived from the application of Kick’s law in macro-scale indentation hardness testing that a hardness independent of the indentation load exists, which is termed as real hardness or absolute hardness. The term ‘real hardness’ is used for this paper. Technically, the real hardness value could be obtained in microhardness testing only when the ISE is quantitatively evaluated. Along with the increasing applications of microhardness tests, extensive research has been devoted to ISE [4,5,6,7,8,9,10,11]. The causes of ISE were summarised to include strain hardening, the barrier load to initiate plastic deformation, elastic recovery in unloading, the activation energy of dislocation motion and dislocation pinning [2]. Nix and Gao explained the ISE by introducing a concept of geometrically necessary dislocations produced during an indentation process to distinguish from the intrinsic dislocations existing in the material [8]. More recently, Liu attributed the ISE to the experimental error and the linear indenting elastic resistance [3]. Sarangi reported the significant contribution of coarse grain size to ISE [12]. 

Meanwhile, several numerical models have been developed to provide quantitative correlations between the indentation load, indent diagonal length and the ISE-independent hardness (real hardness). Hays and Kendall supposed that a constant elastic resistance *W* exists in the indentation hardness testing when an indentation load *P* is applied. Consequently, the effective indentation load should be equal to (*P*-*W*). Then, the Hays-Kendall model can be written as in Equation (5) when it is applied to Vickers microhardness [13], where the units of the loads *P* and *W*, the real hardness *H_0_*, and the diagonal length *d* are kgf, kgf/mm^2^ and mm, respectively. Li and Bradt modified the Hays–Kendall model by proposing a linear resistance to the diagonal length *d* [7]. Then, the Li–Bradt model is written, as shown in Equation (6). Bull and co-researchers set up a polynomial *P*-*d* relationship, as shown in Equation (7) [4]. Nix and Gao made a different approach to the explanation of the ISE by attributing it to the generation of a strain gradient and geometrically necessary dislocations in the indentation-induced deformation volume, to distinguish from the existing statistically distributed dislocations [8]. The Nix-Gao model is shown in Equation (8), where *H* and *h* are the measured hardness and indentation depth, *H_0_* stands for the real hardness and h^*^ is a constant related to the indenter geometry, the elastic shear modulus and hardening property of the tested material [14]. In Vickers indentation, the Nix-Gao is also written as Equation (9) for the linear relationship between the indentation depth *h* and the indent diagonal length *d*.

(5) $$P=W+\frac{H_{0}}{1.8544\;}\cdot \;d^{2}$$

(6) $$P=a\;\cdot \;d+\frac{H_{0}}{1.8544\;}\cdot \;d^{2}$$

(7) $$P=W+a\;\cdot \;d+\frac{H_{0}}{1.8544\;}\cdot \;d^{2}$$

(8) $$H=H_{0}\;\cdot \;\sqrt{1+\frac{h^{*}}{h}}$$

(9) $$H=H_{0}\;\cdot \;\sqrt{1+\frac{d^{*}}{d}}$$

Microhardness tests are widely employed to assess the strain-hardening behaviour in many circumstances, such as shot peening and pre-rolling [15,16,17], cyclic rolling fatigue in rails and bearings [18,19,20] and various wear processes [21,22,23]. Most of the obtained microhardness properties, however, did not present the real hardness properties, because the significant influence of the ISE was not considered. It may cause scientific misunderstanding if the remarkable difference between the measured ISE-affected microhardness and the real ISE-free hardness is ignored. Similar problems also exist in the evaluation of hard coatings, in which microhardness and nano-indentation are still the major methods [24,25]. The established models have enabled microhardness testing to measure the real hardness and hardening behaviour of various materials, such as annealed and strain-hardened stainless steels [2,26], irradiation-hardened vanadium alloys [27], ceramics and glass [28], steels of different chemical compositions and hardened conditions [12,29,30], and other materials [31,32]. It has been noted however, that few cases were conducted on materials having dimensions down to micro-scales, such as particles, fibers, thin films and micro-scale engineering surfaces. 

The aim of this research was to demonstrate the reliability of several established theoretical models in the calculation of the real hardness. Consequently, the real hardness can be measured without using large indentation loads, whereas such a method is in high demand for small-dimension materials. An austenitic high-Mn Hadfield steel, which shows good plasticity and strong strain hardening capacity, was selected for this research. Several strain-hardened samples were tested using Vickers indentation at small loads from 0.01 to 1.0 kgf. The measured hardness data were analysed using Meyer’s power law, to investigate their performance in assessing the ISE behaviour. In addition to the determination of the real hardness, the effect of plastic strain on the ISE behaviour was investigated. 

## 2. Experimental Method and Data Analysis

### 2.1. The Sample Materials

Firstly, three specimens were cut from a used off-track railway turnout, which was an austenitic Hadfield steel having compositions (in wt%) of C 0.98, Mn 13.05, Si 0.51, P 0.02, Cr 0.03 and Fe in balance. The first specimen was cut from the rail top, which presented an extreme condition of plastic deformation, hardening and embrittlement of Hadfield steel, because of the confirmed occurrence of extensive spalling and delamination failure. 

The second specimen was cut from a tensile-tested bar to present the state of ultimate tensile elongation. The tested bar was one of the three tensile bars taken from the bottom part of the used turnout to determine the tensile properties of the bulk Hadfield steel. The tensile bar recorded an elongation of 47% and an area reduction ratio of 35%, in addition to other mechanical properties of Young’s modulus of 202 GPa, the ultimate tensile strength of 773 MPa and the yielding strength of 358 MPa. 

The third specimen was cut from the bottom part of the turnout. It presented the as-manufactured and non-deformed bulk steel. The Hadfield steel, according to its microstructure characteristics, was treated by the conventional austenisation and subsequent water-quenching to obtain the single-phase austenitic microstructure. 

In addition to the three specimens having different plastic deformation scales, another group of samples were selected to carry out two case studies using the newly selected methodology. The first sample was a cross-sectioned rail surface of the used turnout, in which the surface failure was expected to be associated with a gradient of subsurface strain hardening. The second case comprised an austenitic Hadfield steel, a pearlitic rail steel, and a quenched martensitic 300 M steel, each including the bulk material and a sliding worn surface. This case aimed to investigate the effect of sliding wear and microstructure on real hardness and ISA behaviour. The worn surfaces were produced in a reciprocating dry-sliding ball-on-disc wear test using a UBM-3 multi-functional tribometer (Center for Tribology, Inc. (CERT), Campbell, CA, USA). The wear test was performed using a WC counterpart ball, an applied load of 5 kgf, a reciprocating length of 8 mm, a nominal sliding speed of 20 mm/s to produce 47 sliding passes per minutes and a total sliding time of 400 min per sample. More details of the wear tests have been recently published elsewhere [33,34].

The samples were metallographically ground and polished to a mirror finish. Such procedure was also applied to the curving rail top sample, where a flat band of approximately 4 mm in width was created to facilitate the microhardness measurement. For the worn surfaces, pre-cleaning was applied using soft and wet tissue to uncover the metallic worn surface by removing the attached loose wear debris. 

### 2.2. Methods of Microhardness Testing and Characterisation

**Figure 1:**
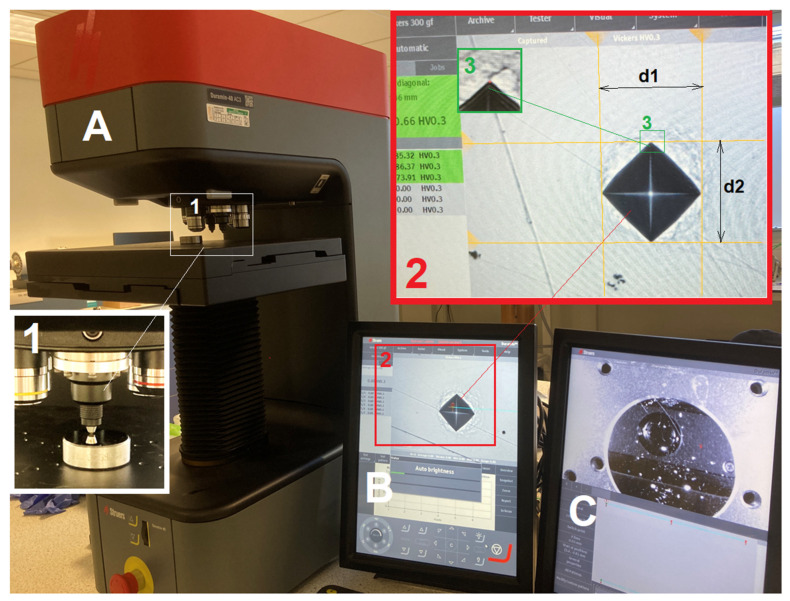
Photographic illustration of the hardness tester employed in the experiments: part (**A**) is the main body of the tester; parts (**B**,**C**) are the monitors of the control panel. The insert 1 in Part (**A**) shows details of the indenter, a sample under indentation and the optical lens for observation; The inserts 2 shows the measurement of the two diagonal lengths, *d*_1_ and *d*_2_; and the insert 3 shows the precise positioning of an indent tip point.

A hardness tester, Struers Duramin-40 AC3 (STRUERS APS, Ballerup, Denmark), was employed. Figure 1 shows a photographic view of the tester and the processing in measuring the diagonal length of an indent. Careful focusing was manually applied to every indent to be measured in order to ensure the precision of the measurement. 

Prior to the testing, an indirect verification experiment was undertaken using a UKAS calibration steel block of known microhardness of HV_0.3_ 385.2, which showed a measured hardness of HV_0.3_ 381.8 ± 8.0 corresponding to the indent diagonal length of 0.03811 ± 0.00040 mm. Accordingly, the relative deviation of the hardness and diagonal length are −0.5% and 0.3%, respectively. The latter is less than 1.5% as specified by the standard ISO 6507-2. The repeatability and percent bias were calculated, according to the equations defined in the standard ISO 6507-2, to be 5.2% and −0.54%, respectively. These values are below the criteria of the repeatability (8.0%) and percent bias (7.01%) as specified by the standard ISO 6507-2, respectively. 

The indentation loads were selected to be from 0.01 to 1.0 kgf with a selected dwelling time of 10 s. The diagonal lengths of each obtained Vickers indent were measured using the attached optical microscope. At each condition, six indentations were made, whereas the spacing between two indents was not less than three times the resultant diagonal length. 

The samples were characterised using scanning electron microscopy (SEM) and X-ray diffraction (XRD) analysis. A FEI Nova 200 FEG-SEM instrument (FEI Europe BV, Eindhoven, The Netherlands) was employed for the microstructure observation. Prior to SEM observation, the specimens to be analysed were metallographically ground and polished to a mirror-finish, and chemically etched using a 2% nital etchant. An Empyrean X-ray diffractometer (Malvern Panalytical BV, Almelo, The Netherlands) with Co-K_α_ radiation (wavelength *λ* = 0.1789 nm) was employed for the XRD analysis. The XRD scans were conducted using the θ−2θ scan mode, with a step size of 0.026° and a scanning speed of 0.0022°·s^−1^. 

### 2.3. Methods of Data Analyses

#### 2.3.1. Calculations of the Meyer Index *n*, Real Hardness *H*_0_, and ISE Significance Coefficient *η*

On each group of data, the applied loads *P* were plotted versus the resultant diagonal lengths *d*. The *P*–*d* data series was fitted Meyer’s power law, as shown in Equation (4), using the regression analysis function provided in MS Excel. Accordingly, the Meyer index *n* and the constant *A* were obtained. 

Then, the four theoretical models, including the Hays–Kendall model, the Li–Bradt model, the Bull model and the Nix-Gao model, were employed to calculate the real hardness *H_0_*. The calculations were based on Equations (5)–(8), and performed using the regression analysis function provided in MS Excel. Details of the calculation methods are summarized in Table 1. In case of the Hays–Kendall model, for example, the indentation loads {*P*} were plotted versus the square of indent diagonal length {*d*^2^}, followed by a linear regression to deduce the real hardness *H*_0_ and the constant indentation resistance *W*, according to Equation (5). After that, an ISE significance coefficient was defined as *η* = $\frac{H-H_{0}}{H_{0}},$ according to the measured indentation hardness *H* and the real hardness *H*_0_. Thus, a positive or negative *η* value means the presence of ISE, whereas *η* = 0 stands for an ISE-free state. The calculations of the coefficient are also provided in Table 1. 

#### 2.3.2. Prediction of Hardness Using the Theoretical Models

The four theoretical models, as provided in Equations (5)–(8), were employed to predict the indentation hardness values for a series of indentation load. For the Hays–Kendall model, Equation (5) can be used to project the indent diagonal length *d* for each applied indentation load *P*. Then, Equation (10) is developed from Equations (2) and (5) to project the hardness values *H*_1_ and *H*_2_, where *H*_1_ is the hardness value determined from the load *P* and diagonal *d* using Equation (3), and *H*_2_ is the hardness value determined by the related theoretical model. When both *H*_1_ and *H*_2_ vary with the diagonal *d*, there should be only one *d* value which satisfies both Equations (2) and (9), i.e., making *H*_2_ = *H*_1_. Then, the projected hardness can be determined. Similarly, Equations (11)–(13) are developed to project the hardness values using the Li–Bradt model, Bull model and Nix-Gao model, respectively.

(10) $$H_{1}=1854.4\;\cdot \;\frac{P}{d^{2}};\;H_{2}=H_{0}+\frac{1.8544\cdot W}{d^{2}}$$

(11) $$H_{1}=1854.4\;\cdot \;\frac{P}{d^{2}};\;H_{2}=H_{0}+\frac{1.8544\cdot a}{d}$$

(12) $$H_{1}=1854.4\;\cdot \;\frac{P}{d^{2}};\;H_{2}=H_{0}+\frac{1.8544\cdot \left(a_{0}+a_{1}\cdot d\right)}{d^{2}}$$

(13) $$H_{1}=1854.4\;\cdot \;\frac{P}{d^{2}};\;H_{2}=H_{0}\;\cdot \;\sqrt{1+\frac{d^{*}}{d}}$$

## 3. Results

### 3.1. Effect of Straining on The Microstructure of the Austenitic Hadfield Steel

**Figure 2:**
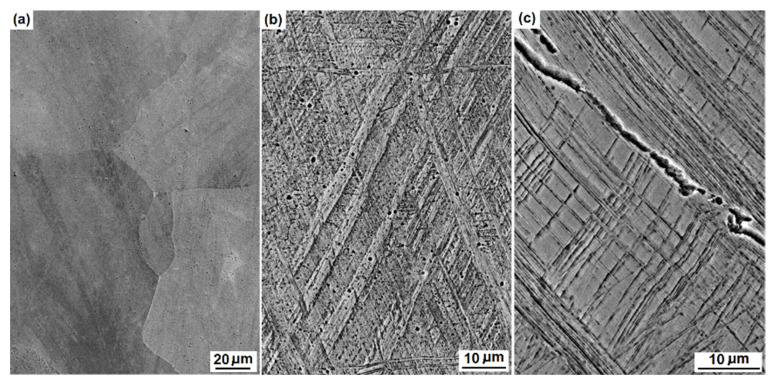
The microstructure of Hadfield steel under various strained conditions: (**a**) the bulk steel; (**b**) the 47%-elongated tensile bar; and (**c**) the worn top of turnout.

**Figure 3:**
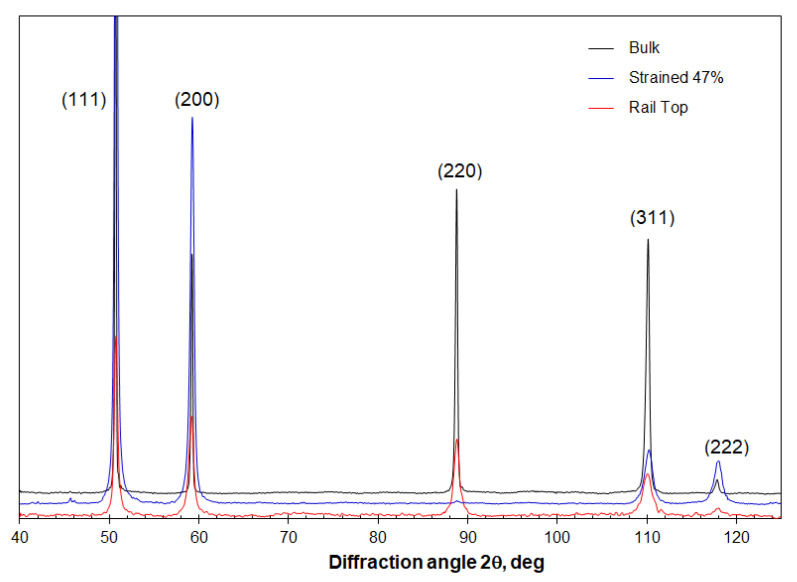
The X-ray diffraction curves of the Hadfield steel under various strained conditions, showing different broadening of the austenite diffraction peaks.

The microstructure of the three Hadfield steel samples is shown in Figure 2. The bulk steel exhibits coarse grains of single austenitic phase with clear grain boundaries, as shown in Figure 2a. Figure 2b shows the microstructure of the tensile-strained bar, showing bi-directional deformation twins. Figure 2c shows the microstructure approximately 45 μm beneath the rail top. The steel exhibited densely distributed deformation twins and a crack following the twinning direction. These mechanical twins suggest a rolling-induced strain hardening of Hadfield steels [19,20]. Figure 3 shows the XRD patterns of the three samples, in which the austenite peaks (111), (200), (220), (311) and (222) indicate their single-phase crystalline structure. The diffraction peaks of the tensile strained and rail top samples are significantly broader than those of the bulk steel. The SEM and XRD characterisation confirm significant straining of the tensile sample and the rail top as compared to the bulk steel. 

### 3.2. The Vickers Microhardness Property Determined by Indentation

**Figure 4:**
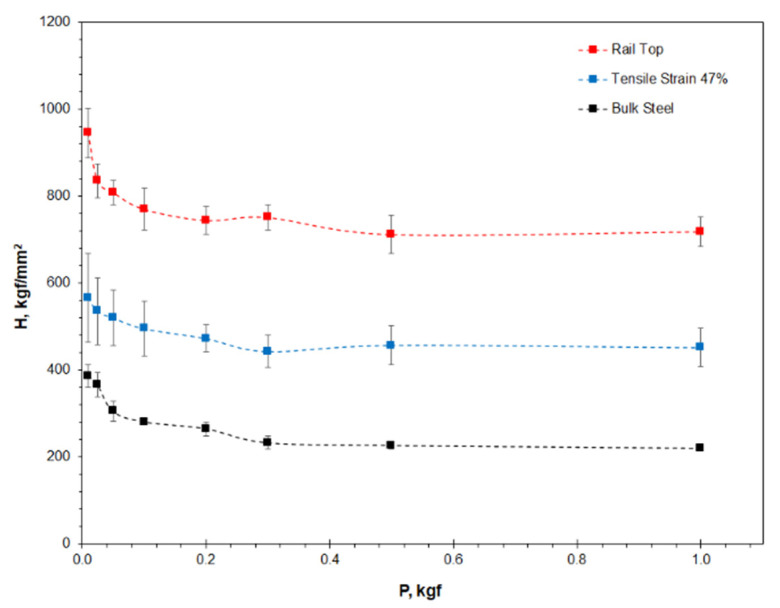
The microhardness properties of the three Hadfield steel samples plotted versus the applied indentation load.

Figure 4 and Table 2 shows the microhardness properties of the three Hadfield steel samples measured under indentation loads from 0.01 to 1.0 kgf. The rail top has the highest hardness, followed by the strained tensile bar and the unstrained bulk steel. Obviously, both the tensile bar and the rail surface were remarkably strain hardened. The cracking, delamination and spalling failures observed on the rail top suggests that it had reached an extreme stain-hardening state. The tensile bar only accounts for approximately 50% of the hardening scale of the top rail. 

In Figure 4, the microhardness depends highly on the indentation load especially at the low-load end, which confirms the ISE characteristics. In addition, it was noted in the experiments that the bulk steel hardness was measured with very small deviation (between 5 and 26), which should be attributed to the good homogeneity of the solutioning-treated steel. On the other hand, the two strained samples exhibited strong deviation of the measured microhardness (between 28 and 102). The data scattering suggests the heterogeneous nature of the strain hardening, which is evidenced in the non-uniform distributions of the deformation bands as shown in Figure 2b,c.

### 3.3. The Real Hardness and ISE Significance Determined Using the Theoretical Models

**Figure 5:**
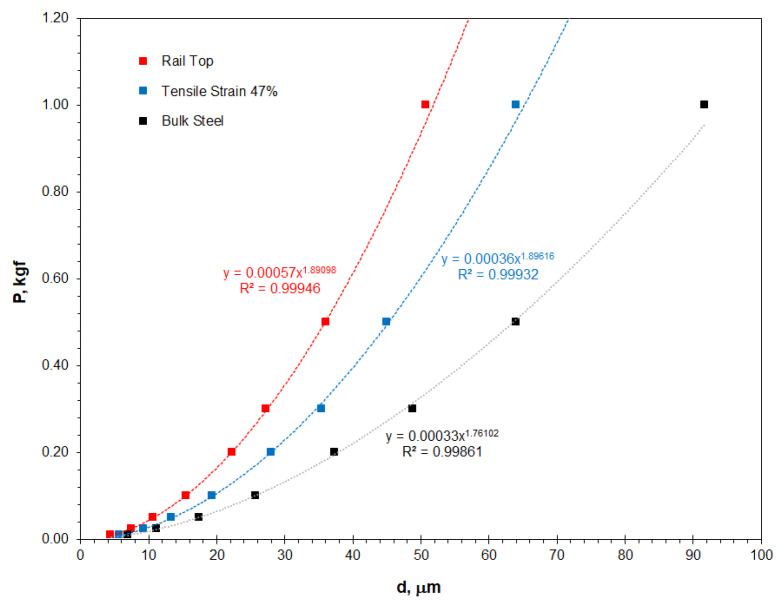
Meyer’s P~d profiles of the Hadfield steel, showing effect of plastic strain on the power law relationship.

Figure 5 shows the resultant indent diagonal lengths plotted versus the applied indentation loads. The three plots are fitted to Meyer’s power law, referring to Equation (4), with an extremely high relevance factor, *R*^2^ ≥ 0.999. The values of Meyer index *n* are not equal to, but less than, 2, suggesting positive ISE. The constant *A* also varies between the three samples, and the rail top shows the maximum value because of its highest hardness as shown in Figure 4. 

**Figure 6:**
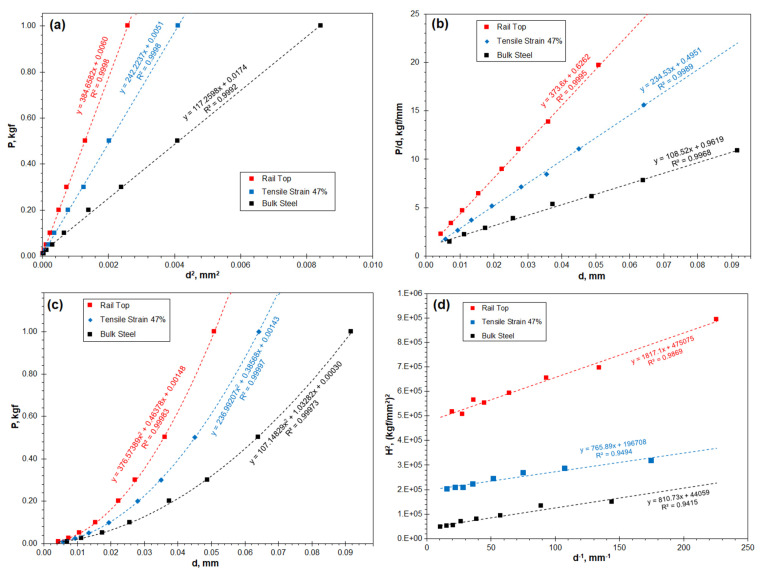
Linear regression plots to calculate the real hardness of the three Hadfield steel samples using the theoretical models: (**a**) using the Hays–Kendall model; (**b**) using the Li–Bradt model; (**c**) using the Bull model; and (**d**) using the Nix-Gao model.

Figure 6 shows linear regression charts of the three samples using the Hays–Kendall, Li–Bradt, Bull and Nix-Gao models. The high relevance factor *R*^2^ suggests that the four models have good feasibility in analysing the real hardness and the ISE phenomenon. The real hardness of the three samples is listed in Table 3, in which the results show good consistency. Compared to the low hardness of the bulk steel, the tensile sample reached a high hardness of 443 kgf/mm^2^. The rail top gained a hardness of 697 kgf/mm^2^, which can be considered as the maximum achievable hardening when the associated embrittlement became sufficient to trigger spalling and delamination failures. Considering the high relevance factor *R*^2,^ as shown in Figure 6, an attempt was made to undertake the same modelling analyses using the hardness data obtained in the microhardness range, i.e., from 0.01 to 0.2 kgf. The determined real hardness values are shown in Table 4. Comparing Table 3 and Table 4, the real hardness of the two strained samples shows little change, despite a slight increase in the relative deviation. The results suggest that the real indentation hardness can be measured with reasonably high accuracy by using the indentation loads not exceeding 0.2 kgf. 

**Figure 7:**
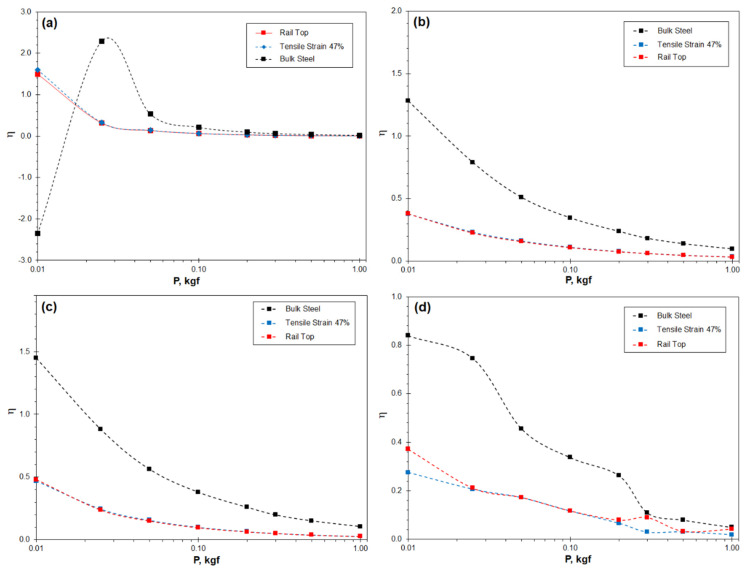
The ISE significance coefficient η of the three Hadfield steel samples determined using the theoretical models: (**a**) using the Hays−Kendall model; (**b**) using the Li−Bradt model; (**c**) using the Li−Bradt model; and (**d**) using the Nix−Gao model.

Figure 7 shows the ISE significance coefficient *η* was determined using the four theoretical models as a function of the indentation load. Firstly, high *η* values are obtained when the indentation loads are in the microhardness range, i.e., 0.01–0.2 kgf, whereas the coefficient approaches to zero when the indentation load is higher. Such behaviours reveal high sensitivity of indentation hardness in the microhardness range. Secondly, the bulk steel exhibits the most significant ISE. In contrast, the two pre-strained samples show lower *η* values being very close to each other, indicative of greatly decreased ISE significance. 

Figure 7 also illustrates the difference ISE significance arising from the four theoretical models. The Hays−Kendall and Nix−Gao models show the highest and lowest *η* values, respectively. The *η* values obtained from the Bull and Li–Bradt models are intermediate. 

### 3.4. Compatibility of the Theoretically Projected Hardness to the Measured Hardness 

**Figure 8:**
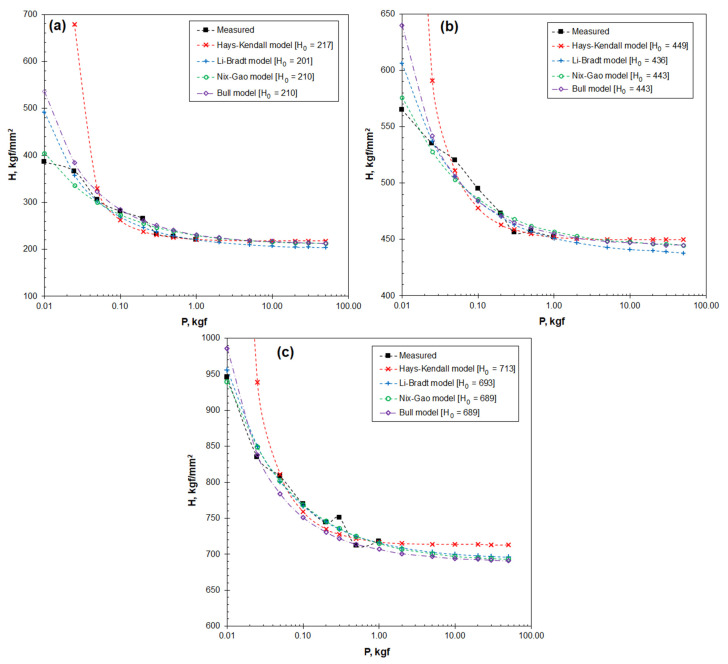
Comparison between the measured hardness and the hardness projected using the theoretical models: (**a**) the bulk steel; (**b**) the 47%−elongated tensile bar; and (**c**) the rail top.

Figure 8 shows the results of the compatibility study using the four employed theoretical models. The scattering of the projected hardness value with respect to the measured hardness value has been quantified to show the performance of the four theoretical models. The results are summarized in Table 5. The hardness values projected from the four models are plotted for a range of indentation loads from 0.01 to 50 kgf, to be compared to the experimentally measured hardness values. The four models behave differently in projecting hardness at the lower indentation loads, i.e., when the load is smaller than 0.05 kgf. In Figure 8a, the Nix−Gao model is the one best fitting the measured hardness of the bulk steel. The Li−Bradt model also exhibits good compatibility except at the lowest indentation load of 0.01 kgf. The Hays–Kendall model shows the worst compatibility to the measured hardness, followed by the Bull model. 

Figure 8b,c shows the compatibility of the strained samples. The Hays–Kendall model again shows the worst compatibility in the two cases. The Li–Bradt model and the Bull model show intermediate mismatches. Whereas no theoretical model fits absolutely to the measured hardness values, the Nix-Gao model shows the minimum mismatch between the projected and the measured hardness. In Table 5, the Nix-Gao model outperforms the other models by showing the lowest scattering in the indentation load range of 0.02–0.2 kgf, e.g., Δ*H* = 9 ± 6 for the tensile bar. Based on the analysis, the Nix-Gao model was subsequently selected in the two reported cases to measure the real hardness and ISE behaviour of strained Hadfield steels. 

### 3.5. Case Studies: Initial Applications of the Established Method 

#### 3.5.1. The Microhardness Properties of the Worn Surfaces of Steels Having Different Microstructure

**Figure 9:**
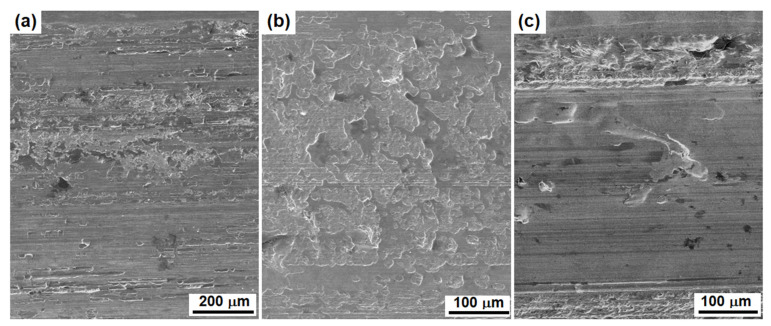
SEM micrographs showing the worn surfaces: (**a**) the M18 austenitic Hadfield steel; (**b**) the pearlitic rail steel; and (**c**) the martensitic 300 M steel.

After the Nix-Gao model was selected out of the four models, initial applications were undertaken in two cases. The first case was to characterise the wear-induced hardening of three steels having different microstructure, since sliding between solid surfaces is known to cause severe plastic deformation. The three steels included an austenitic Hadfield steel (Mn18), a pearlitic rail steel (P71) and a martensitic high-strength steel (300 M). Figure 9 shows the worn surfaces analysed. More details of the microstructure and wear failure mechanisms have been published elsewhere [33,34].

**Figure 10:**
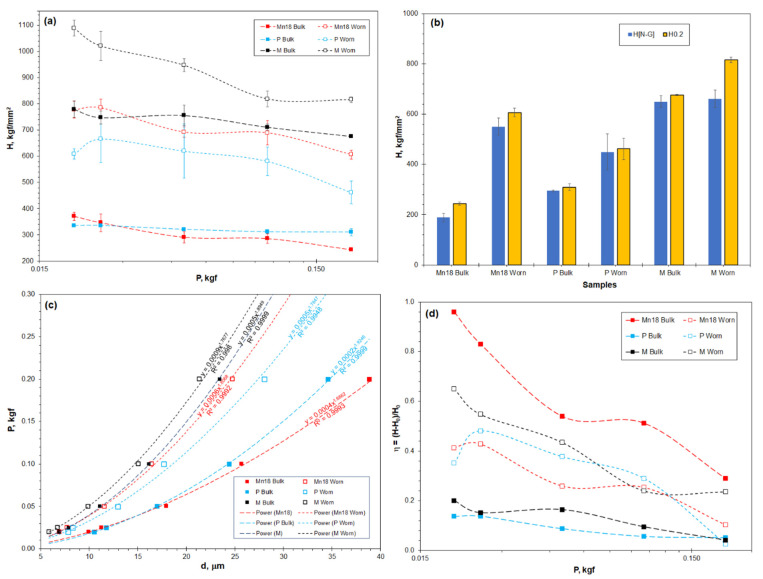
The hardness and ISE properties of the three different microstructure steels and their worn surfaces: (**a**) the measured microhardness plotted versus indentation load; (**b**) the real hardness (*H*_N-G_ determined from the Nix-Gao model) as compared to the microhardness at the indentation load 0.2 kgf (*H*_0.2_); (**c**) Meyer’s power profiles; and (**d**) the ISE significance coefficient *η*.

Figure 10a shows the measured microhardness of the three pairs of worn surface and bulk steel measured plotted versus the applied indentation loads from 0.02 to 0.2 kgf. Figure 10b shows the real hardness *H*_N-G_, determined using the Nix-Gao model, and the microhardness *H*_0.2_ of the six samples. The real hardness *H*_N-G_ differs from the microhardness *H*_0.2_ in all cases, due to the influence of the ISE. Comparing to the bulk steel, the wear-induced hardening of the austenitic Hadfield steel is the most significant, followed by the pearlitic rail steel. The 300M steel also exhibits a certain scale of hardening, suggesting that the applied sliding wear was able to make further hardening to the martensite microstructure. 

In Figure 10c, the indentations of the six samples obey Meyer’s power law with very high relevance factor *R*^2^. The samples, however, show quite different values of Meyer’s index *n*. The Hadfield steel worn surface shows a higher index of 1.81 than the bulk steel (*n* = 1.69), suggesting decreased ISE significance of the worn surface. The worn surfaces of the pearlitic and martensitic steels show a decreased index as compared to the bulk samples, suggesting increased ISE significance of the worn surfaces.

The ISE significance coefficient *η*, calculated using the equations provided in Table 1, is shown in Figure 10d. ISE has been found to exist in all cases, whereas the *η* coefficient is strongly dependent on the material and the indentation load. Firstly, the *η* coefficient shows strong load dependence, which is increasingly significant at low loads. Secondly, the austenitic steel shows much stronger ISE than the pearlitic and martensitic steels. Thirdly, the sliding wear caused variations of the *η* coefficient. The *η* value of the austenitic Hadfield steel is greatly decreased in its worn surface. In contrast, the worn surfaces of both the pearlitic and martensitic steels show increased *η* values compared to the bulk steels. 

#### 3.5.2. The Subsurface Hardness Profile of the Worn Hadfield Steel Turnout

**Figure 11:**
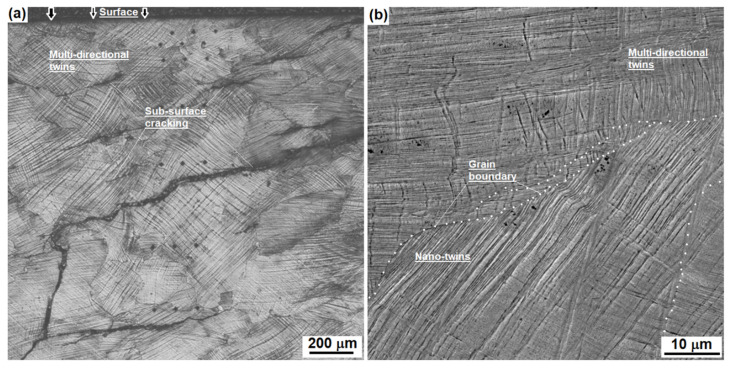
Cross-sectional microstructure of the worn Hadfield steel turnout: (**a**) an optical image showing deformation bands and cracks from the rail top at a certain depth; and (**b**) a SEM image showing different orientations of mechanical twins beside a grain boundary.

The second case was to measure the strain-hardening behaviour in various depth of the worn Hadfield steel turnout. Figure 11 shows the cross-sectional microstructure at a certain depth, indicating the developed cracks and heterogeneous distribution of deformation bands. 

**Figure 12:**
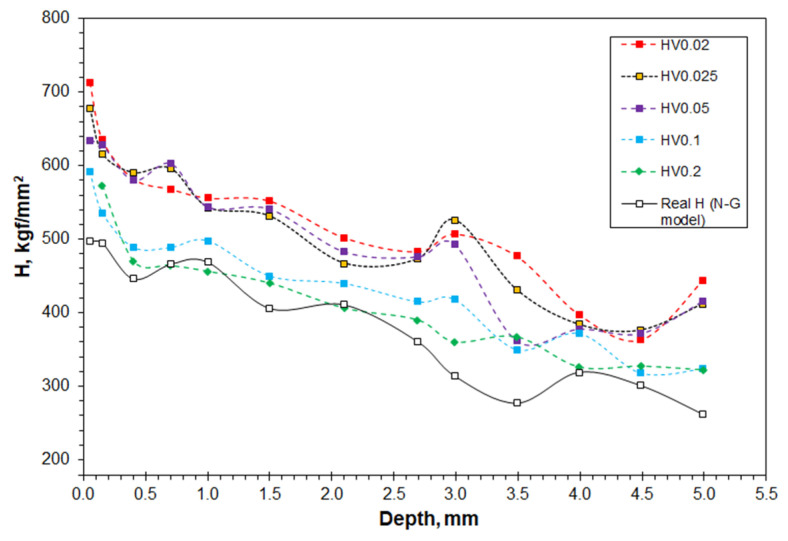
Depth profiles of microhardness and real hardness of the worn Hadfield steel turnout.

Figure 12 shows the microhardness and real hardness properties in a range of depth positions beneath the turnout worn surface. The microhardness measured at every depth position is dependent on the indentation load, confirming pronounced ISE phenomenon. The highest values were measured at the lowest load of 0.02 kgf in most depths. The highest microhardness was measured in close vicinity of the rail top to be higher than 700 kgf/mm^2^. In contrast, the microhardness values measured at the highest indentation load of 0.2 kgf are the lowest. The real hardness at each depth is even lower than the values of the microhardness. 

Nevertheless, based on the real hardness analysis, it is feasible to compare the strain-hardening statue of Hadfield steel caused under different loading conditions. The real hardness reached about 500 kgf/mm^2^, following by decreasing values, with increasing depth. Within the depth of 1.5–2.0 mm, the real hardness is higher than 400 kgf/mm^2^, being lower than the real hardness of the rail top (689 kgf/mm^2^, Figure 8c) and the worn surface (550 kgf/mm^2^, Figure 10b), but higher than or equivalent to the hardness obtained on the strained tensile bar (443 kgf/mm^2^, Figure 8b). 

## 4. Discussion

### 4.1. Selection of Theoretical Models to Determine the ISE-Independent Real Hardness

A major contribution of this paper is the establishment of the combined Vickers microhardness testing and the Nix-Gao model to determine the real hardness *H*_0_ of micro-scale volumes where macro-scale indentation is impossible. Comparing between the real hardness and the conventional Vickers microhardness, a drawback of the latter is obvious. The microhardness property has been shown to depend strongly on the applied independent load (Figure 4, Figure 10a and Figure 12). The real hardness, on the other hand, approached the hardness measured at macro-scale loads, which makes it independent to the indentation load (Table 1, Table 2, Table 3 and Table 4). Because of the ISE and its dependence on indentation load and on material characteristics, large uncertainties are expected in a comparative study of microhardness properties of different materials, or when the properties are obtained at different indentation conditions. These uncertainties have been overcome by measuring the real hardness, as is demonstrated in the two cases (Figure 10 and Figure 12).

Several theoretical models have been developed to calculate the real hardness [2]. A major effort of the current research is the quantitative assessments of the four selected theoretical models, for the feasibility of these models in micro-scale hardness measurements was rarely evaluated. The assessments include, firstly, the definition of the ISE significance coefficient *η* to illustrate its dependence on the indentation load and material’s state, as shown in Table 1 and, subsequently, in Figure 7 and Figure 10d. Secondly, the hardness values projected using the four theoretical models have been compared to the experimentally measured hardness values, as shown in Equations (10)–(13) and, subsequently, in Figure 8. This comparison has shown the compatibility of the models to the real measurements in the applied indentation loads. These assessments have helped the establishment of the Nix-Gao model (Equation (9)). 

The current research reveals that the four theoretical models all exhibit good relevance in regression analyses, as shown in Figure 6 and Table 3. These results are consistent with the results of other researchers [3,12,26,27,28,29,30,31]. A new finding of current research is that good compatibility of the theoretical models with the measured hardness does not always exist when the indentation load was low, e.g., lower than 0.05 kgf, as shown in Figure 8. The load dependence can be presented using the newly defined *η* coefficient, as shown in Figure 7. 

Therefore, the current research differs from previous research, such as those results published by other researchers [3,12,14,27,31], in that the selection of a suitable model depends not only on the general relevance of a model to experimental measurements, but also on its precision in treating the strong ISE at low indentation loads. The recommendation of the Nix-Gao model was based on its outstanding performance at the lower end of the applied indentation loads. On the other hand, however, the other models were less recommended for micro-scale measurement due to the pronounced *η* values. This conclusion differs from the published work where these models exhibited good performance [2,26,30,32]. 

### 4.2. The Origin of ISE

The current research reveals the significant existence of ISE, as shown in Figure 4 and Figure 8a. Previously, the origin of ISE was investigated either by considering the elastic-plastic indentation mechanics [2,3,7], or by attributing it to the generation of crystalline defects, i.e., the so-called geometrically necessary dislocations, in contrast to the statistically distributed dislocations [8]. Despite the fact that these have contributed to the understanding of the hardness property, as well as its dependence on the applied indentation load, the current research considers that the origin of ISE is from the definition of indentation hardness. In other words, the ISE phenomenon is attributed to the difference between Kick’s law and Meyer’s law, as shown in Equations (1) and (2). Originally, the definition of indentation hardness derived from Kick’s law, the square relationship between *P* and *d*^2^. In fact, Kick’s law is a special case of Meyer’s power law, in which the index *n* is assumed to be 2, whereas in most cases *n* ≠ 2 has been experimentally confirmed. 

The present experimental results obtained for several steels show that the square relationship between the indentation load *P* and the indent diagonal *d* obeys Meyers power law, having Meyer’s at *n* ≠ 2. On the other hand, the relationship between *P* and *d* does not satisfy the square relationship, as defined by Kick’s law. Therefore, Kick’s law must be revised and adapted to the material under examination. Thus, whereas Meyer’s power law was previously described as an empirical equation [2], it actually implies the existence of ISE by showing the non-square relationship between *P* and *d*^2^. This is true especially when the applied indentation loads fell into the microhardness range. 

### 4.3. Effect of Mechanical Straining on the ISE and Real Hardness of Hadfield Steels

The strong strain-hardening capacity of austenitic Hadfield steels has been well known for a long time. Whereas the hardening was characterised mostly using microhardness tests, the ISE factor was previously ignored in most comparative studies [15,18,19,20,33,34,35,36,37,38]. Consequently, the relations between ISE, plastic deformation severity and strain-hardening properties were precluded from interpretation of the microhardness properties. 

The current research has revealed the strong influence of plastic deformation on the hardness, strain-hardening and ISE significance of the Hadfield steel. Table 6 shows a summary of the ISE-related properties. The strain-hardening ratio was defined as the relative variation of the hardness measured or calculated on the strain-hardened sample, as compared to the hardness of the non-deformed bulk sample, which is similar to the definition provided in literature [21]. Firstly, the plastic deformation influenced the *P*–*d* correlation, i.e., Meyer’s power law, significantly, as shown in Figure 5. Severe plastic deformation of the Hadfield steel made Meyer’s index *n* increase significantly from 1.761 to 1.891 and 1.896. The ISE significance coefficient *η* can be seen to depend strongly on the straining condition, as shown in detail in Figure 7. The non-deformed bulk steel exhibited a stronger ISE significance than the strained samples. In other words, the plastic deformation remarkably reduced the ISE significance. Meanwhile, the constant *A* also changed substantially, from 0.00033 of the bulk steel to 0.00057, which explained why the extremely strain-hardened rail top exhibited the highest real hardness and severe embrittlement. 

Secondly, an advantage of the real hardness estimation is that both the real hardness and the real strain-hardening ratio can be obtained without using indentation loads in macro scale. Table 6 shows the difference between the load-dependent microhardness and the load-independent hardness of the three samples. It is noted that the real hardness and the deduced strain-hardening ratio are both comparable to those properties obtained at high indentation load, i.e., 1 kgf in this case. On the other hand, the strain-hardening ratio derived from the Vickers hardness varies with the indentation load. For example, the rail top has hardness values of HV_0.01_ 945 and HV_1_ 718, corresponding to the indentation loads of 0.01 and 1 kgf, respectively, Accordingly, the strain-hardening ratio against the non-deformed bulk steel is 2.4 and 3.3, respectively, whereas the average strain-hardening ratio calculated from the microhardness range of 0.01–0.2 kgf is 2.6. Meanwhile, another strained sample, the tensile bar, shows strain-hardening ratios different from the rail top sample. These differences suggest that the experimentally measured strain-hardening severity depends both on the material, i.e., the plastic deformation scale, and on the applied indentation load. The method reported in this paper brings about the feasibility of quantifying the hardness and strain-hardening properties by using microhardness measurements. 

### 4.4. Effect of Microstructure on the ISE Properties

Although the case studies conducted in this research were to verify the effectiveness of the new method in measuring the real hardness of worn surfaces, the experiment results provided in Figure 10 also show the strong influence of microstructure on the ISE properties of tested steels. Table 7 summarises Meyer’s index *n* and the ISE significance coefficient *η* of the three steels and their worn surfaces. The austenitic steel exhibits the strongest ISE, as shown in the largest *η* value and the smallest *n* value. In contrast, the pearlitic and martensitic microstructures both exhibit significantly lower ISE, as featured by the higher *n* values and the lower *η* values. These results reveal that the microstructure and chemical composition have a pivotal influence on the ISE behaviour of steels. The influence would be attributed to the different chemical compositions, crystalline structures and phase constituents of the three steels, although extensive explanation is possible only after further extensive experimental work. 

Moreover, the ISE behaviour of the worn surfaces differs remarkably from the bulk steels. Note that the statistical analyses of the *n* and *η* factors reveal much smaller deviation of the worn surfaces, i.e., 0.02 compared to 0.13 of the index *n*, and 0.07 compared to 0.30 of the *η* coefficient. Obviously, the ISE characteristics of the worn surfaces can be seen to be dominated by the wear-induced plastic deformation. Nevertheless, because of the strong dependence of the ISE properties on the chemical compositions, microstructure and plastic deformation, it is important to determine the load-independent hardness property in order to precisely evaluate the hardening behaviour of materials. 

## 5. Conclusions

Through Vickers indentation hardness testing on plastic-strained austenitic Hadfield steel samples, we investigated the origin of the ISE phenomenon. A method to measure the real hardness of small-scale materials was proposed after an extensive assessment of several theoretical models including the Hays–Kendall model, Li–Bradt model, Bull model and Nix-Gao model. The following conclusions can be made.

- The real hardness of small-scale materials can be determined by Vickers microhardness indentation and subsequent analysis using the Nix-Gao model. The origin of ISE derives from the mismatch between the experimentally determined *P* ~ *d* relationship and Kick’s law (*P* = *A* · *d*^2^). Within the limitation of the present experimental activity, the results suggest that Kick’s law should be replaced by Meyer’s power law (*P* = *A* · *d^n^*) with *n* < 2.
- Because of the ISE, indentation hardness measured under small loads does not present the real hardness property and the real strain-hardening ratio. The plastically strained samples exhibited not only strong work hardening, but also different ISE significance, as compared to the non-deformed bulk steel. The bulk steel retained the lowest *A* and *n* values, obtaining the lowest hardness and the strongest ISE significance. The strained samples showed increased an *A* value, with the *n* approaching 2, indicating higher hardness and lower ISE significance, respectively. The sample experienced the extreme plastic straining showed the highest *A* value, reaching a real hardness of 689 kgf/mm^2^.
- When the indentation loads were in macro scale, the four theoretical models show good precision in calculating the real hardness and in predicting the Vickers hardness values at various loads. When the indentation loads were in micro scale, the Nix-Gao model outperformed the other theoretical models in these calculations.

## Figures and Tables

**Table 1:** The regression analysis to calculate the real hardness ***H*_0_** and ISE significance coefficient ***η***.

Models and Equations		$\mathrm{Regression}:\;\;Y=a_{0}+a_{1}\cdot X$		*H*_0_	ISE Parameter	*η*
		*Y*	*X*			
Hays-Kendall	*P* = *W* + $\frac{H_{0}}{1.8544}$ · *d*^2^	*P*	*d* ^2^	1.8544 · $a_{1}$	*W* = $a_{0}$	$\frac{W}{P-W}$
Li-Bradt	*P* = *a* · *d* + $\frac{H_{0}}{1.8544}$ · *d*^2^ $\frac{P}{d}$ = *a* + $\frac{H_{0}}{1.8544}\cdot d$	$\frac{P}{d}$	*d*	1.8544 · *a*_1_	*a* = $a_{0}$	$\frac{1.8544\cdot a}{d\cdot H_{0}}$
Bull	*P* = *W* + *a* · *d* + $\frac{H_{0}}{1.8544}$ · *d*^2^ (*Y* = *a*_0_ + *a*_1_ · *X* + *a*_2_ · *X*^2^)	*P*	*d*	1.8544 · *a*_2_	*W* = $a_{0}$ *a* = $a_{1}$	$\frac{1.8544}{d\cdot H_{0}}$ · (*a*_1_ + $\frac{a_{0}}{d}$)
Nix-Gao	*H* = *H*_0_ · $\sqrt{1+\frac{d^{*}}{d}}$ $H^{2}=H_{0}^{2}$ +$H_{0}^{2}$·$\frac{d^{*}}{d}$	$H^{2}$	$d^{-1}$	$\sqrt{a_{0}}$	$d^{*}$ = $\frac{a_{1}}{a_{0}}$	$\sqrt{1+\frac{d^{*}}{d}}$ − 1

**Table 2:** The average hardness and deviation of the three Hadfield steel samples measured at various loads.

Sample	Indentation Load, kgf							
	0.01	0.025	0.05	0.1	0.2	0.3	0.5	1.0
Rail Top	945 ± 57	835 ± 39	808 ± 28	770 ± 48	744 ± 32	751 ± 29	712 ± 43	718 ± 34
Tensile Strain 47%	565 ± 102	535 ± 76	520 ± 64	495 ± 62	473 ± 31	443 ± 37	457 ± 44	452 ± 44
Bulk Steel	386 ± 26	366 ± 28	305 ± 23	281 ± 5	265 ± 16	233 ± 15	227 ± 9	220 ± 6

**Table 3:** Determination of the real hardness (*H*_0_, in kgf/mm^2^) of the strained Hadfield steel using the theoretical models.

Theoretical Models	Bulk Steel	Tensile Bar	Rail Top
Hays-Kendall	217 ± 3	449 ± 2	713 ± 4
Li-Bradt	201 ± 5	436 ± 3	693 ± 7
Bull	210 ± 13	443 ± 7	689 ± 7
Nix-Gao	210 ± 13	443 ± 7	689 ± 7
Average	210	443	697
deviation	3.1%	1.2%	1.9%

**Table 4:** Determination of the real hardness of the strained Hadfield steel using indentation loads from 0.01 to 0.2 kgf.

Theoretical Models	Bulk Steel	Tensile Bar	Rail Top
Hays-Kendall	258 ± 4	467 ± 6	734 ± 6
Li-Bradt	232 ± 8	448 ± 8	696 ± 5
Bull	226 ± 9	414 ± 6	680 ± 9
Nix-Gao	234 ± 21	459 ± 10	682 ± 9
Average	238	447	698
deviation	5.6%	5.2%	3.6%

**Table 5:** The absolute difference (Δ*H*) between the projected and measured hardness in the load range 0.02–0.2 kgf. (Δ*H* = *H*_measured_ − *H*_projected_ for *H*_projected_ < *H*_measured_; Δ*H* = *H*_projected_ − *H*_measured_ for *H*_projected_ > *H*_measured_).

Models		Bulk Steel	Rail Top	Tensile Bar
Hays-Kendall	Range	19–313	3–104	9–56
	mean ± dev	96 ± 145	32 ± 48	23 ± 22
Li-Bradt	Range	2–106	1–15	2–41
	mean ± dev	29 ± 43	7 ± 6	14 ± 16
Nix-Gao	Range	5–30	1–14	0–17
	mean ± dev	14 ± 10	6 ± 5	9 ± 6
Bull	Range	4–150	3–41	2–75
	mean ± dev	39 ± 63	20 ± 14	22 ± 30

**Table 6:** Comparison of the ISE-related properties of strain-hardened Hadfield steel samples.

Properties		Rail Top	Tensile Bar	Bulk Steel
Meyer’s constant *A* and index *n*	*A*	0.00057	0.00036	0.00033
	*n*	1.891	1.896	1.761
ISE significance coefficient *η*	Mean value	0.14	0.11	0.36
	Range	0.04–0.37	0.02–0.27	0.05–0.84
Hardness *H*	*H*_0_	689	443	210
	HV_0.01_	945	565	386
	HV_1_	718	452	220
Strain-hardening ratio ($\frac{H-H_{bulk}}{H_{bulk}}$ · 100%)	By *H*_N-G_	3.3	2.1	1.0
	By HV_0.01_	2.4	1.5	1.0
	By HV_0.01–0.2_	2.6	1.6	1.0
	By HV_1_	3.3	2.1	1.0

**Table 7:** Comparison of the ISE-related properties of different microstructure steels.

Steels	Meyer’s Index *n*		ISE Significance Coefficient *η*	
	Bulk	Worn	Bulk	Worn
Austenitic Mn18	1.69	1.81	0.63	0.29
Pearlitic rail	1.92	1.78	0.10	0.31
Martensitic 300M	1.89	1.77	0.13	0.42
Mean	1.83	1.79	0.29	0.34
Deviation	0.13	0.02	0.30	0.07

## Data Availability

No further data is available.
